# Inferior vena cava thrombosis as a possible cause of nephrotic-range proteinuria: two case reports

**DOI:** 10.1186/s13256-021-03132-6

**Published:** 2021-11-25

**Authors:** Yana Apostolova, Patricia Mehier, Salah D. Qanadli, Menno Pruijm

**Affiliations:** 1 Department of Internal Medicine, University Hospital of Lausanne and University of Lausanne (CHUV), Rue du Bugnon 46, 1011 Lausanne, Switzerland; 2 Service of Nephrology, Riviera-Chablais Hospital, Rennaz, Switzerland; 3 Department of Radiology, Interventional Radiology, University Hospital of Lausanne and University of Lausanne, Lausanne, Switzerland; 4 Service of Nephrology, University Hospital of Lausanne and University of Lausanne, Lausanne, Switzerland

**Keywords:** Nephrotic syndrome, Inferior vena cava, Orthostatic proteinuria

## Abstract

**Background:**

Nephrotic-range proteinuria is a common reason for nephrological consultation in clinical practice. The differential diagnosis is wide, and generally focuses on different forms of glomerulonephritis, but other causes should not be overlooked, as illustrated in this article.

**Case presentations:**

We report two female patients with nephrotic-range proteinuria. In the first case, a 46 year old Caucasian patient who suffered from extreme obesity (Body mass index (BMI) 77 kg/m^2^), acute kidney injury and nephrotic-range proteinuria were discovered during an emergency consultation for acute abdominal pain. The second patient (aged 52, also Caucasian) developed stage 4 chronic kidney disease and nephrotic proteinuria (protein/creatinine ratio 1821 g/mol) after accidental rupture of the inferior vena cava during a gastric bypass operation. On split-urine collection, both had a much higher degree of proteinuria during the day than during the night, compatible with orthostatic proteinuria. At further work-up, inferior vena cava thrombosis was diagnosed in both patients, whereas renal veins were patent.

**Discussion:**

After simple anticoagulation in the first case, and anticoagulation plus endovascular recanalization in the second, there was almost complete resolution of the orthostatic proteinuria and a strong improvement of the estimated glomerular filtration rate in both patients. These cases highlight that nephrotic-range proteinuria can be linked to inferior vena cava thrombosis, and that a split-urine collection may also be very useful in the diagnostic work-up of proteinuria in adults.

## Background

Nephrotic-range proteinuria is a common reason for nephrological referral in clinical practice, and defined as renal protein loss of more than 3.5 g/24 hours. It can occur in the absence or presence or presence of edema and hypoalbuminemia (< 35 g/l). The latter situation is known as nephrotic syndrome [1].

Nephrotic-range proteinuria with or without nephrotic syndrome can be caused by a wide variety of diseases. Frequent causes include different forms of glomerulonephritis (minimal change disease, membranous nephropathy, and focal segmental glomerulosclerosis). Among the secondary causes are systemic diseases such as diabetes mellitus, amyloidosis, lupus, and different types of vasculitis. The initial work-up of proteinuria includes extensive blood and urine testing, and is often followed by a kidney biopsy if the cause remains uncertain. Urine tests include dipstick analysis, microscopic analysis of the urinary sediment, and quantification of the degree of proteinuria. For quantification, random urine samples or 24 hour urine collections are used. In random urine samples, the ratio of total protein-to-creatinine ratio (expressed as g protein/mol creatinine) is measured as a proxy of 24 hours proteinuria. Split day and night urine collections are only rarely performed, except in children where they can point towards underlying orthostatic proteinuria.

Orthostatic proteinuria is defined as an abnormally large amount of protein excreted in urine when the patient is in an upright position, versus a normal or absent amount in the supine position. This condition is usually benign and of uncertain etiology, although it is sometimes linked to a compromised return of blood in the left renal vein caused by compression by the superior mesenteric artery (so called nutcracker phenomenon) [2]. Orthostatic proteinuria affects mostly children and adolescents, and often resolves without any treatment [3].

The following two cases demonstrate that split-urine collection might also be useful in the work-up of nephrotic-range proteinuria in adults and point towards an unusual cause of proteinuria.

## Case presentation

### Case 1

A 46 year old female Caucasian patient consulted the emergency department for right-sided flank pain. The patient had history of type 2 diabetes mellitus, obstructive sleep apnea syndrome, and schizoaffective disorder. She was treated with oral antidiabetic drugs (metformin and sitagliptin) and antipsychotics (valproate and amisulpride). She also suffered from morbid obesity and weighed 207 kg, with a height of 1.64 m (body mass index of 77 kg/m^2^).

**Fig. 1:**
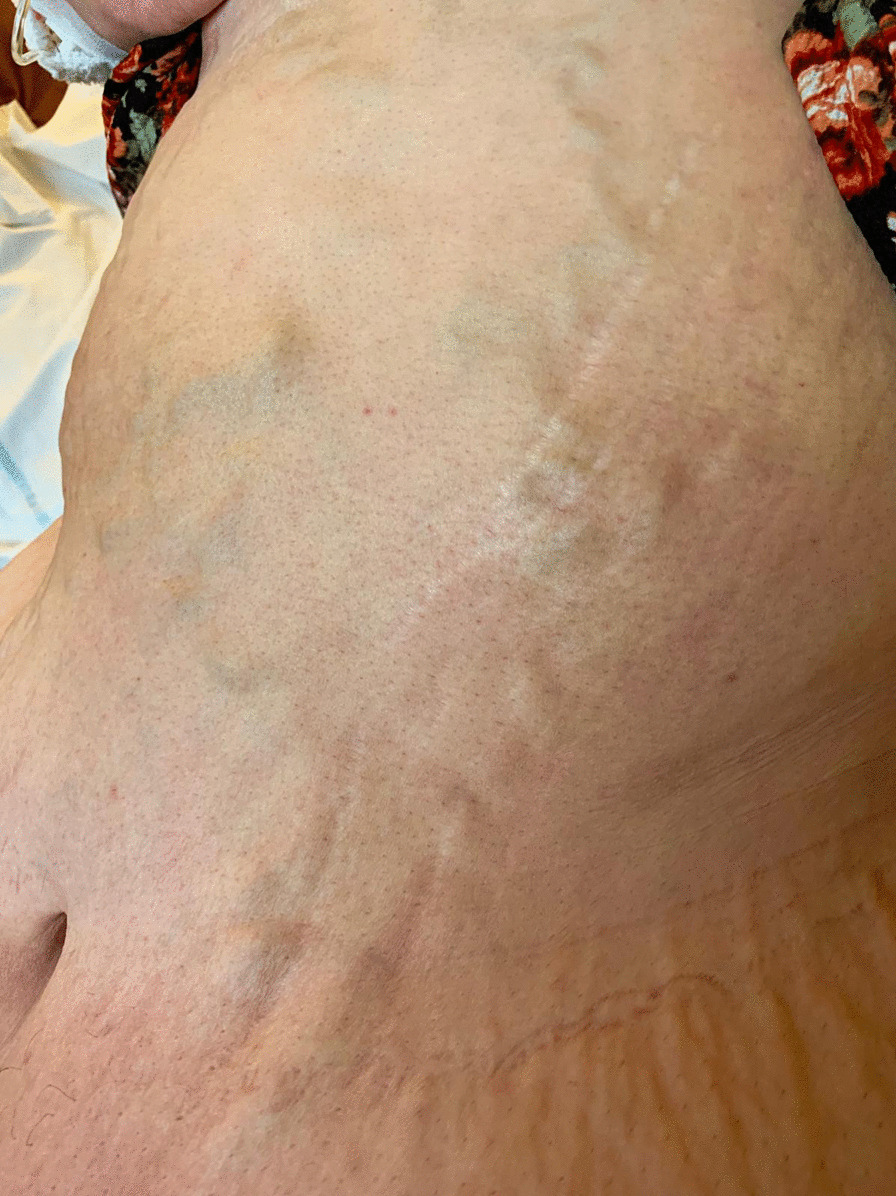
Extreme obesity with overhanging abdomen and distended superficial abdominal veins at first presentation of patient 1

The clinical examination revealed distension of superficial veins of the abdominal wall (see Fig. 1) and tenderness over the right abdominal flank. She also had chronic lymphedema of the legs.

The laboratory values on admission showed an increased level of creatinine (115 μmol/l, normal range: 44–80 µmol/l), compared with a baseline value of 80 μmol/l. The rest of the laboratory values were normal [albumin 39 g/l (*N*= 35–52 g/l)], liver function tests were all within normal range, leukocytes 7.0 Giga/l (*N*= 4.0–10.0 G/l), hemoglobin 141 g/l (*N*= 117–157 g/l), and thrombocytes 168 G/l (*N*= 150–350 G/l)), except C-reactive protein level that was slightly elevated at 30 mg/l (*N* < 10 mg/l).

Urine dipstick analysis did not show leukocytes or erythrocytes, but the presence of protein. A random urine spot sample confirmed the presence of proteinuria, with a urine protein-to-creatinine ratio (uPCR) of 4485 g/mol (*N* < 10), corresponding to an estimated daily protein excretion rate of 44 g/day; of note, 1 g/mol corresponds to 1 mg/mmol [4–6]. The urine protein electrophoresis showed albumin, but no paraproteins.

The patient refused hospitalization but accepted an outpatient nephrology consultation that took place 2 days later. The creatinine level had gone up 48 hours later, from 115 to 178 μmol/l. To our surprise, a significant decrease of the proteinuria was noted, with a protein/creatinine ratio of 194 g/mol. Of interest, the nephrology consultation took place in the morning, whereas the emergency visit took place during the evening, as seen in orthostatic proteinuria.

Although the initial diagnostic hypothesis for the rapid renal function loss included glomerulonephritis (FSGS, minimal change or membranous glomerulonephritis) or diabetic nephropathy, the collateral abdominal circulation and the orthostatic proteinuria raised the suspicion of an alternative cause, such as obstruction of the renal veins or inferior vena cava.

**Fig. 2:**
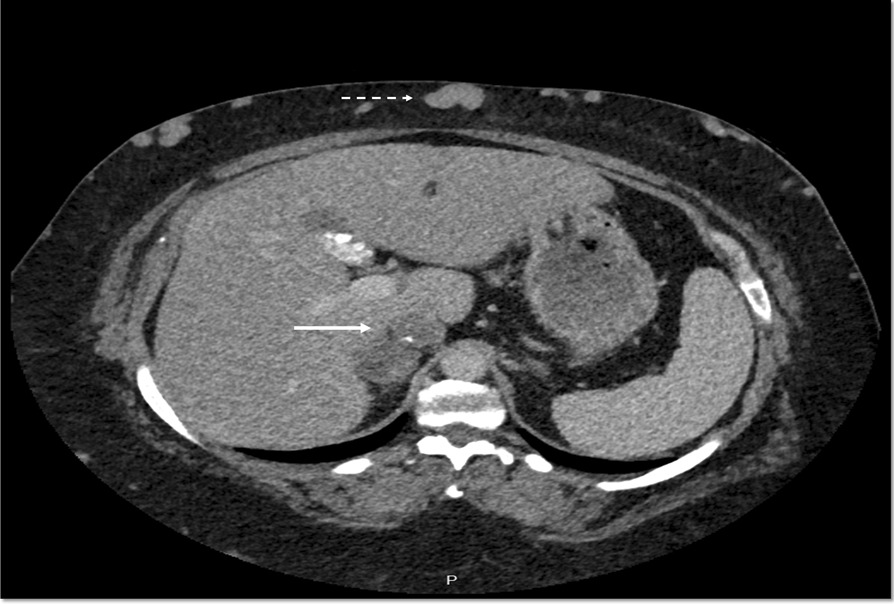
Contrast-enhanced CT scan of patient 1: IVC thrombosis (bottom arrow) and collateral abdominal veins (top, notched arrow) are clearly visible

To rule out thrombosis and to assess the feasibility of kidney biopsy, a computed tomography (CT) scan with iodine contrast was performed. The CT showed extensive thrombosis of the suprarenal inferior vena cava without thrombosis in the renal veins and portal vein (Fig. 2). This time, the patient accepted hospitalization and she was admitted to the hospital for intravenous heparin. The immunological work-up [antinuclear factor (ANF), antineutrophil cytoplasmic (ANCA), antiglomerular basal membrane (anti-GBM), and antiphospholipase A2 (anti-PLA2) antibodies] was negative. During hospitalization, split urine collection was performed and showed elevated uPCR values during the day (595 g/mol) compared with the night (early morning sample: 45 g/mol), see Table 1 for details.

**Table 1:** Summary of the laboratory results of patient one

Date	Urine protein/creatinine (g/mol, *N* < 10)		Urine albumin/creatinine (mg/mmol, *N* < 3)		Creatinine (μmol/l, *N*= 44–80)
	Day	Night	Day	Night	
Emergency presentation, day 1	4485		2910		134
Day 3, nephrology outpatient clinic		194		119	178
Day 4, hospitalization	1498		935		153
Day 6, split urine collection; anticoagulation started	595^a^	45	335^a^	15.3	
Day 26, nephrology outpatient clinic	194		109		82

^a^Result of 24 hour urine collection

The renal function improved progressively, and distention of the superficial abdominal collateral veins diminished. A kidney biopsy was not performed for technical reasons. The patient was discharged on oral anticoagulation with anti-vitamin K (acenocoumarol). The follow-up CT at 3 months confirmed a partial recanalization of the inferior vena cava, and laboratory testing showed a further decrease in uPCR (45 g/mol in a random afternoon sample).

### Case 2

This 52-year-old Caucasian female was referred to our nephrology clinic for investigation of nephrotic range proteinuria and a recent rise in creatinine levels. Her medical history included a left nephrectomy that had been performed 10 years earlier for pyonephrosis secondary to obstructive nephrolithiasis. Seventeen months prior to the current consultation, she had undergone a surgical intervention abroad; the goal was a gastric bypass, but the surgery was interrupted because of an early accidental rupture of the inferior vena cava and gall bladder. A cholecystectomy was performed, as well as primary repair of the blood vessel. A few days after the surgery, a control CT scan showed suprarenal inferior vena cava (IVC) thrombosis without renal vein thrombosis. Oral anticoagulation (apixaban) was initiated and continued for 6 months. Five months before the actual consultation, she underwent a control abdominal CT-scan. This scan showed persistent suprarenal IVC thrombosis that now expanded in the extra-hepatic veins; besides, a collateral circulation had developed via the azygos veins. Renal function worsened progressively, with a rise in creatinine from 126 to 248 μmol/l, and spot urinary analysis showed nephrotic-range proteinuria, which motivated nephrological consultation.

Despite worsening renal function, the patient was asymptomatic. At clinical examination, her blood pressure was 117/83 mmHg while taking three antihypertensive drugs [a calcium inhibitor (amlodipine), renin-angiotensin system (RAS) inhibitor (irbesartan), and a beta blocker (bisoprolol)]. Heart and lung auscultation were normal and she did not have malleolar edema. Her BMI was 39 kg/m^2^ (weight 89 kg, height 1.51 m). There was no hematuria or leukocyturia in the urinary sediment. In a random urine sample, the uPCR was 1821 g/mol, corresponding to an estimated protein excretion of 18 g/day. Albumin accounted for 64% of the total proteinuria. Basic immunological work-up (antinuclear factor, antiphospholipase A2 antibodies, antineutrophil cytoplasmic antibodies, anti-double-stranded-DNA antibodies, antiglomerular basal membrane antibodies, complement factor C3 and C4) and serologic tests for HIV and hepatitis B and C were all negative. Circulating albumin, C-reactive protein (CRP), electrolytes, and liver function tests were all within normal range, and there were no paraproteins. The initial working diagnosis was glomerulonephritis due to minimal change disease or membranous nephropathy. However, the risk of performing a kidney biopsy was considered too high in the setting of a single kidney, the venous collateral circulation, and the apixaban that could not be easily stopped.

As alternative diagnosis, and in analogy with the first case, we hypothesized that the extensive IVC thrombosis could be partly responsible. To further examine this possibility, we obtained a split urine sample. There was a significant difference in urine protein secretion between the morning (uPCR of 1.5 g/mol) and the afternoon sample (uPCR 1153 g/mol, Table 2). This result suggested that the renal venous congestion was more pronounced in the upright position.

**Table 2:** Summary of the laboratory results of patient two

Date	Urine protein/creatinine ratio (g/mol, *N* < 10)		Urine albumin/creatinine ratio (mg/mmol, *N* < 3)		Creatinine (μmol/l, *N*= 44–80)
	Standing	Supine	Standing	Supine	
First nephrological consultation, 1 year after operation	1821		1175		164
Follow up consultation, split urine collection	1153	15	767	4.2	133
One month after recanalization	15^a^	0.74^a^	8^a^	3^a^	53^a^

^a^Results of 24 hour urine collection

**Fig. 3:**
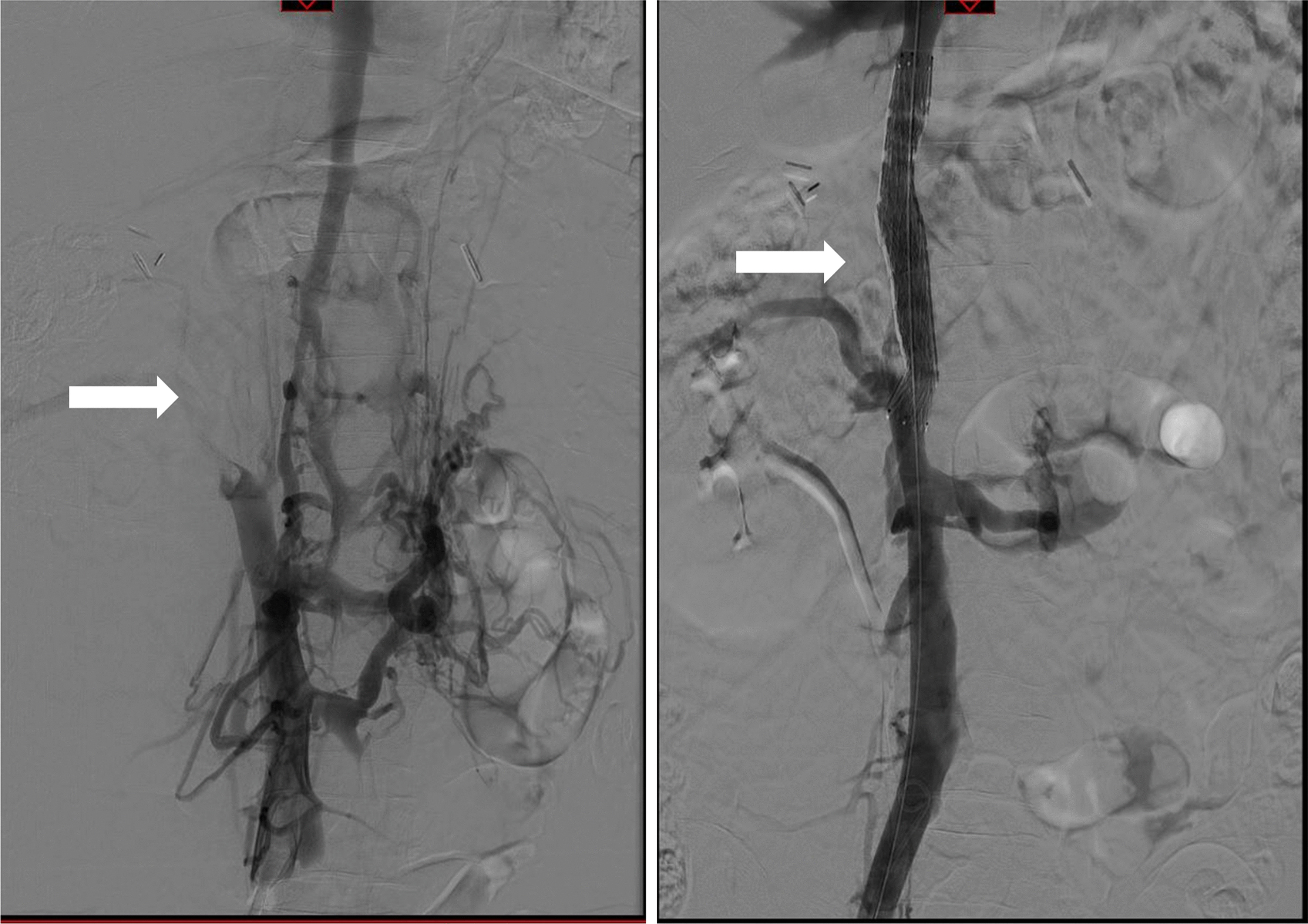
Angiography before (left) and after (right) revascularisation of the IVC in patient 2. Localization of the IVC thrombosis is shown with an arrow (left figure), as is the stent (right figure). Note the disappearance of the collateral circulation around the IVC and the left kidney. Angiography before and end-vascular revascularisation procedure with recanalization and stunting if the IVC in patient two

As a proof of principle, the patient underwent a successful endovascular revascularization procedure with recanalization and stenting of the IVC (Fig. 3). Hereafter, renal function recovered completely and the proteinuria disappeared, without any other change in therapy (Table 2).

## Discussion

In this article, we describe the clinical presentation, diagnostic procedures, and treatment of two patients with IVC thrombosis. Both patients presented with nephrotic-range proteinuria of orthostatic nature that diminished or even disappeared after systemic anticoagulation and/or recanalization of the IVC, respectively, suggesting that IVC thrombosis was the most likely cause of their proteinuria.

Although renal vein thrombosis is a well-known complication of nephrotic syndrome in patients with glomerulonephritis, the opposite (thrombosis as a cause of proteinuria) has rarely been described. As for IVC thrombosis, to the best of our knowledge, only one article (published in 1962) has previously reported that isolated thrombosis of the IVC can cause nephrotic range proteinuria [7]. However, at the time CT scans were not available, and associated renal vein thrombosis was not excluded with certainty. Renal vein thrombosis in association with IVC thrombosis has been reported in several articles as a cause of nephrotic syndrome [8–12]. All reported cases improved after anticoagulation.

The diagnostic clue to the presence of a possible IVC thrombosis was the presence of orthostatic proteinuria in both patients.

Classically, orthostatic proteinuria points towards an underlying nutcracker syndrome, especially in adolescents, although it can also be encountered in adults [3]. The nutcracker syndrome is characterized by the entrapment of the left renal vein between the aorta and the superior mesenteric artery. It is hypothesized that this leads to venous hypertension and ruptures in the septa between the small veins and collecting systems of the renal fornix, thus explaining the hematuria. Besides, venous hypertension may lead to a release of norepinephrine and angiotensin II, especially in the upright position when the renal vein is further compressed. As a consequence, transglomerular pressure and glomerular permeability for proteins increase, explaining the orthostatic proteinuria [13, 14]. The CT scans in our patients did not show any signs of underlying nutcracker syndrome. Moreover, this syndrome is often associated with lower BMI [2], whereas our patients were obese.

Nevertheless, a similar mechanism may explain the orthostatic proteinuria observed in our cases. IVC thrombosis may increase the pressure in the renal veins and also increases the transglomerular pressure, ultimately leading to proteinuria and reduced glomerular filtration rate. Obesity may reinforce this phenomenon. Indeed, the pressure gradient between the thoracic and abdominal vena cava and the intra-abdominal pressure are higher in obese patients than in controls, and increase further in the standing position [15]. This phenomenon may explain the fluctuations in proteinuria observed in our patients, despite the fixed IVC thrombosis.

Although this is an attractive hypothesis, we have no proof that these mechanisms were indeed present in the present cases, as renal venous pressures were not measured.

After IVC thrombosis, collateral circulations will ultimately develop, especially via the azygos system. This could diminish (but possibly not normalize) the pressure in the renal veins [16]. The collateral, abdominal venous circulation offered an important clue to the underlying diagnosis of IVC thrombosis in the first patient. Of interest, the collateral circulation quickly disappeared after recanalization of her IVC.

A major limitation in our case reports is the lack of renal biopsies, and we can therefore not exclude the possibility that underlying glomerulonephritis was present in one or both patients. For technical reasons (extreme obesity in patient one and a single kidney in patient two on anticoagulation), renal biopsies were not performed. However, immunological work-up was negative in both cases, and urinary sediments were inactive. Besides, the abrupt reductions in proteinuria after anticoagulation (heparin followed by acenocoumarol) in the first case and endovascular recanalization in the second are strong arguments for a causal link between IVC thrombosis and the nephrotic range proteinuria. Neither of our patients received immunosuppressive treatment, but still had normal renal function during the follow-up, 2 years after the first event.

The cause of IVC thrombosis in the second case was straightforward, as it was a direct complication of a surgical intervention. In the first case, its etiology is less certain. IVC thrombosis is an underdiagnosed pathology with high morbidity and mortality. The IVC thrombosis is often associated with malignancies owing to their prothrombotic properties or direct invasion of the vessel wall (typically renal cell carcinoma). Thrombophilia-related conditions such as antiphospholipid antibody syndrome, hormone replacement therapy, pregnancy, and chronic inflammatory conditions have also been associated with IVC thrombosis. An overview of the possible causes of IVC thrombosis is presented in Table 3 [17]. None of these factors were present in the first patient. The most striking feature of the first patient was her extreme obesity (BMI 77 kg/m^2^). Although obesity is not recognized as an isolated cause of IVC thrombosis, we believe that the extreme obesity played a major role in the first patient. First of all, she was largely immobilized as she could hardly move because of her obesity. Secondly, obesity is associated with increased intra-abdominal pressure and reduced venous blood flow velocity, which further increased her thrombotic risk [18].

**Table 3:** Frequent causes of IVC thrombosis according to the underlying mechanism

Endothelial damage	Stasis	Coagulopathy
Endovascular intervention Surgery Abdominal trauma	Dehydration Hypovolemia Obesity Congenital IVC anomalies External compression	Nephrotic syndrome Thrombophilia Factor V Leiden Antiphospholipid syndrome Jak 2 Syndrome protein S/C

Anticoagulation, either by heparin, vitamin K antagonists, direct Xa inhibitors, or a combination of these molecules, is the mainstay of treatment for patients with IVC thrombosis. To the best of our knowledge, there are no comparative studies among these different molecules, and the choice should therefore, in our opinion, be based on local expertise. Adjunctive therapeutic modalities may be useful in selected patients, depending on the acuity of their presentation. For example, in patients with acute (< 14 days) or sub-acute (15–28 days) presentation who are not at high risk for bleeding, catheter-directed thrombolysis has been successfully applied [19]. Whereas those with chronic presentation (> 28 days) may benefit from percutaneous transluminal angioplasty (PTA), and stenting alone remains uncertain. To the best of our knowledge, only one study assessed the efficacy and long-term prognosis of endovascular management [20]. In this Indian study of 12 cases of chronic IVC thrombosis, all the cases had an immediate and long-term (follow-up of 13 years) procedural success (recanalization +/− stenting), without any complications. Only one case had restenosis, which was successfully managed by stenting. No data were provided on proteinuria in this study.

## Conclusion

The association of isolated IVC thrombosis and proteinuria has been rarely reported in medical literature, but should be considered in patients with orthostatic proteinuria, the presence of a collateral abdominal venous circulation, and a negative immunological work-up for glomerulonephritis. The presented cases suggest that morbid obesity may be a risk factor for this clinical entity, although more studies are necessary to support this hypothesis. A computerized tomography scan with intravenous contrast is the preferred radiological exam to confirm the diagnosis, as Doppler echography may be inconclusive, especially in obese individuals. Patients should be treated with anticoagulation. Whether or not endovascular treatment is indicated should be decided on an individual basis. Endovascular treatment can lead to spectacular improvement in renal function and proteinuria, as shown in this case report.

## Data Availability

Data sharing is not applicable to this article as no datasets were generated or analyzed during the current study.

## Abbreviations

IVC: Inferior vena cava
BMI: Body mass index
NRP: Nephrotic range proteinuria
FSGS: Focal segmental glomerulosclerosis
